# Structural and Optical Properties of Interfacial InSe Thin Film

**DOI:** 10.1021/acsomega.3c06600

**Published:** 2024-02-09

**Authors:** Cansu Emir, Adem Tataroglu, Emre Coşkun, Sema Bilge Ocak

**Affiliations:** † Graduate School of Natural and Applied Sciences, Gazi University, Ankara 06830, Turkey; ‡ Physics Group, Atilim University, Ankara 06830, Turkey; § Department of Physics, Gazi University, Ankara 06830, Turkey; ∥ Department of Physics, Canakkale Onsekiz Mart University, Canakkale 17100, Turkey; ⊥ Department of Physics, Middle East Technical University, Ankara 06830, Turkey

## Abstract

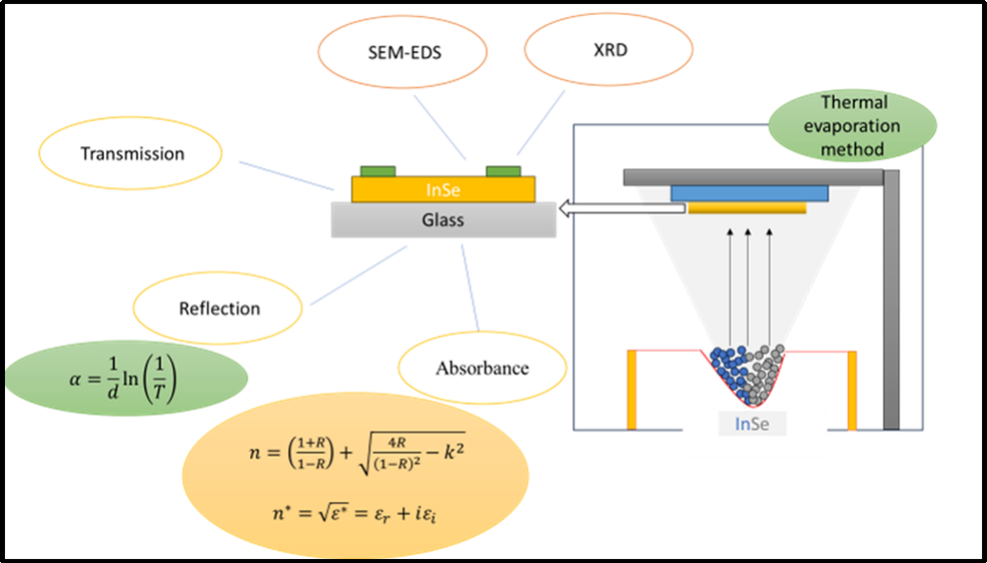

This study presents a comprehensive investigation of the optical and structural characteristics of the indium selenide (InSe) film prepared on a glass substrate. The structural characteristics of the InSe film were analyzed using characterization techniques including X-ray diffraction, scanning electron microscopy, and energy-dispersive X-ray spectroscopy while the UV–vis spectrophotometry method was used in the spectral range between 500 and 1000 nm to examine the optical characteristics. Thus, the UV–vis spectroscopic data were used to extract several optical parameters including extinction coefficient (*k*), optical band gap (*E*_g_), refractive index (*n*), absorption coefficient (α), and optical conductivity (σ_opt_). The optical transition of InSe was found as a direct transition. However, the optical analysis of this study has revealed that the InSe film has the potential to be used in various optoelectronic and photovoltaic applications.

## 1 Introduction

The growing global demand for energy has prompted researchers to explore alternative sources of energy beyond traditional options. Concerns over pollution from conventional energy sources, such as natural gas, petroleum, and nuclear reactors, have intensified the search for cleaner alternatives. In the past decade, particular attention has been directed toward renewable energy forms, with solar energy emerging as a promising candidate due to its affordability, cleanliness, and virtually limitless supply.

Solar energy can be converted into various forms through methods such as photothermal, photochemical, photoelectrochemical, photobiochemical, and photovoltaic. Among these, photovoltaic (PV) and solar cell devices stand out as the cleanest and most efficient means of converting solar energy into electrical power. A solar cell comprises a potential barrier within a semiconductor material capable of separating electrons and holes generated by light absorption within the semiconductor.

Photovoltaic cells exist in two main forms: large-area and thin-film solar cells. Thin film photovoltaic devices offer advantages over their larger counterparts by using smaller amounts of materials and enabling cost-effective processing. Thin films, which are two-dimensional materials formed through the condensation of atoms, molecules, or ions, play a crucial role in various electronic and optical devices.

In recent years, layered semiconductors of the III–VI family, particularly InSe, have garnered significant interest in both thin film and single-crystalline forms due to their unique properties suitable for device applications. InSe layered semiconductors consist of two In and two Se sublayers in each packet with van der Waals-type interlayer (Se–Se) bonding and largely covalent bonding within the layers. This bonding arrangement results in an absence of dangling bonds at the surface, creating an ideal condition for fabricating metal–semiconductor or *p*–*n* heterojunctions. Consequently, interfaces between such layered materials remain unstrained, even with relatively high lattice mismatches.

The exploration of InSe layered semiconductors represents a noteworthy development in photovoltaics, offering potential advancements in thin film technology and contributing to ongoing efforts to harness sustainable and environmentally friendly energy sources.

As one of the post-transition metal chalcogenides (PTMCs), indium selenide (InSe) has a large optical band gap ranging between about 1.25 eV in the bulk form and 2.8 eV in the monolayer form.^1^ Moreover, InSe is a two-dimensional layered material (2D LM) like transition-metal dichalcogenide, graphene, and hexagonal boron nitride. With this feature, InSe is also a promising two-dimensional (2D) semiconductor.^1,2^ Therefore, these semiconductors have drawn significant attention owing to their outstanding electrical and optoelectronic characteristics in the field of nanomaterials and nanodevices. Group III–VI layered semiconductors such as InSe, GaSe, GaS, GaTe, etc. are among the 2D materials. Graphene is an excellent 2D layered structure. Among 2D materials, transition metal dichalcogenides (TMDs) are of great importance for various devices such as photodiodes, transistors, photodetectors, and sensors.^3^ One notable TMD, indium selenide (InSe), has emerged as a promising material with a wide range of practical applications. InSe plays a pivotal role in cutting-edge technologies. InSe’s exceptional semiconducting properties make it an ideal candidate for next-generation electronic devices. Manufacturers are using atomically thin layers of InSe to create ultrathin, high-performance transistors and photodetectors.^4^ These devices are not only incredibly compact but also energy-efficient, enabling faster and more power-efficient smartphones, wearable gadgets, and even flexible electronics for various applications.^5^ Moreover, InSe’s bandgap tunability and strong light-matter interaction make it a vital material for photonics. Researchers have harnessed its unique properties to develop compact and efficient photonic devices such as modulators, light-emitting diodes, and optical sensors.^6^ InSe’s role in these applications can lead to breakthroughs in high-speed data communication and quantum information processing. InSe-based sensors have revolutionized environmental monitoring, healthcare, and security. Its high sensitivity to changes in the surrounding environment allows for the development of ultrasensitive gas sensors, biosensors, and infrared detectors. For example, InSe-based sensors can be used to detect hazardous gases, monitor health parameters, and enhance night vision technology. In addition to these, InSe’s large surface area and excellent electrical conductivity have made it a promising material for energy storage applications. Researchers are exploring InSe in supercapacitors and lithium-ion batteries, aiming to develop high-capacity, long-lasting energy storage solutions for electric vehicles and renewable energy systems.^7^ Consequently, InSe is just one of many TMDs that are reshaping various industries with remarkable properties. As research into TMDs like InSe continues to advance, we can anticipate even more groundbreaking applications in the fields of materials science, electronics, photonics, and beyond.

With its various applications in nanoelectronics and optoelectronics owing to its outstanding properties such as high charge-carrier mobility (10^3^ cm^2^/(V s) at room temperature), band gap covering the visible light region,^3^ slight electron effective mass and great mechanical flexibility, InSe has drawn increasing attention recently. InSe exhibits a direct-to-indirect optical transition. InSe has a significant effect on optical emission.^8^ InSe has covalently bonded Se–In–In-Se layers that are arranged in a hexagonal atomic lattice. van der Waals bonding forces hold the InSe crystal together.^1,2,9−11^ InSe has the potential to be used in several applications of electronic and optoelectronic devices including sensors, photodetectors, field-effect transistors, and solar energy conversion devices.^12−15^ The InSe thin films are produced by using several methods including molecular beam epitaxy, pulsed laser deposition, RF-sputtering, thermal evaporation, electrodeposition, sol–gel, vacuum evaporation, and e-beam technique.^14−17^

In this study, the optical and structural characteristics of the InSe thin film that was deposited by the thermal evaporation method and prepared on a glass substrate were studied in detail. The structural characteristics were examined by using energy-dispersive X-ray spectroscopy (EDX), electron microscopy (SEM), X-ray diffraction (XRD), and scanning electron microscopy (SEM). On the other hand, ultraviolet–visible (UV–vis) spectroscopy was employed to determine the optical constants of the studied material in order to analyze its optical characteristics.

## 2 Experimental Details

**Figure 1:**
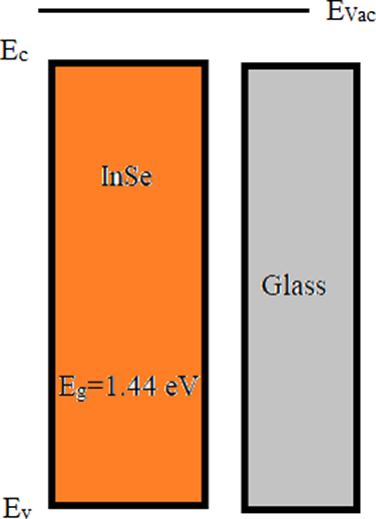
Schematic energy-band diagram for InSe on a glass substrate.

In this research, the thermal evaporation method was employed to deposit the InSe thin film on a glass substrate under a vacuum of 10^–6^ mbar. In this method, the In and Se powders were added to the alumina-coated tungsten boat filament. These glass substrates were rotated during the entire deposition process after they were placed about 25 cm above the evaporation source unit. Rotating the glass substrate allowed us to obtain a uniform thin film. The Inficon XTM/2 quartz crystal transducer-type deposition process monitor was utilized to determine the deposition rate, and it was found to be ∼2–3 Å/s. The Dektak 6 M thickness profilometer was utilized to measure the InSe film’s thickness, and it was found to have a thickness of ∼637 nm. This thickness value was confirmed by an SEM measurement. The annealing of the deposited InSe thin film was performed at various temperatures, and the annealing temperature of 250 °C under a nitrogen atmosphere for 45 min was observed to provide the best crystalline characteristics. The schematic energy band diagram for InSe on a glass substrate is shown in Figure 1.

XRD, EDX, and SEM measurements were conducted to examine the structural characteristics of the deposited thin film studied. The Rigaku MiniFlex diffractometer with CuKα radiation (λ = 0.154 nm) was employed in the XRD method. On the other hand, the Zeiss EVO 15 SEM with an EDX detector was used in the EDS and SEM experiments to reveal the atomic composition of the relevant elements and the surface morphology. The optical characterization of the studied thin films was performed at room temperature. Transmittance, reflectance, and absorption spectra of the InSe film on the glass substrate were recorded by using a HITACHI UV-2600 ultraviolet–visible (UV–vis) Spectrometer by utilizing a wavelength range between 500 and 1000 nm.

## 3 Results and Discussion

### 3.1 Structural Characterization of the InSe Film

**Figure 2:**
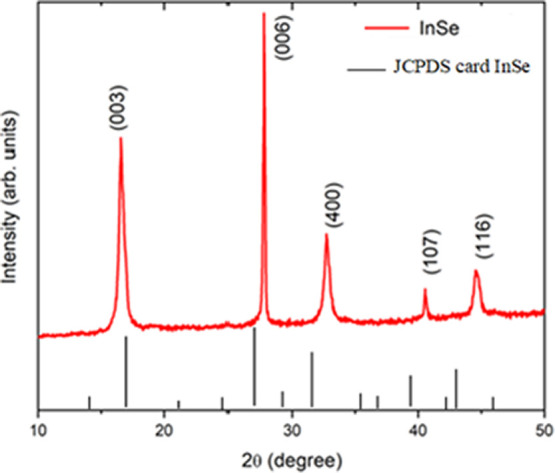
XRD pattern of the studied InSe thin film.

The X-ray diffraction (XRD) method was employed to analyze the crystal structure and orientation of the InSe thin film. Figure 2 presents the XRD pattern of the studied InSe thin film. The observed interplanar spacing, *d*_hkl_, was compared with the data of JCPDS card No. 34-1431. The InSe film exhibits five diffraction peaks at 17.36°, 28.21°, 33.83°, 41.60°, and 45.97° corresponding to the (003), (006), (400), (107), and (116) planes, respectively. The diffraction peaks indicate that the sample is an InSe film.^18−21^ Also, the strongest diffraction peak was observed in the (006) lattice plane. As a result, the XRD pattern indicated that the examined InSe thin film was successfully prepared and had good crystallinity.

**Figure 3:**
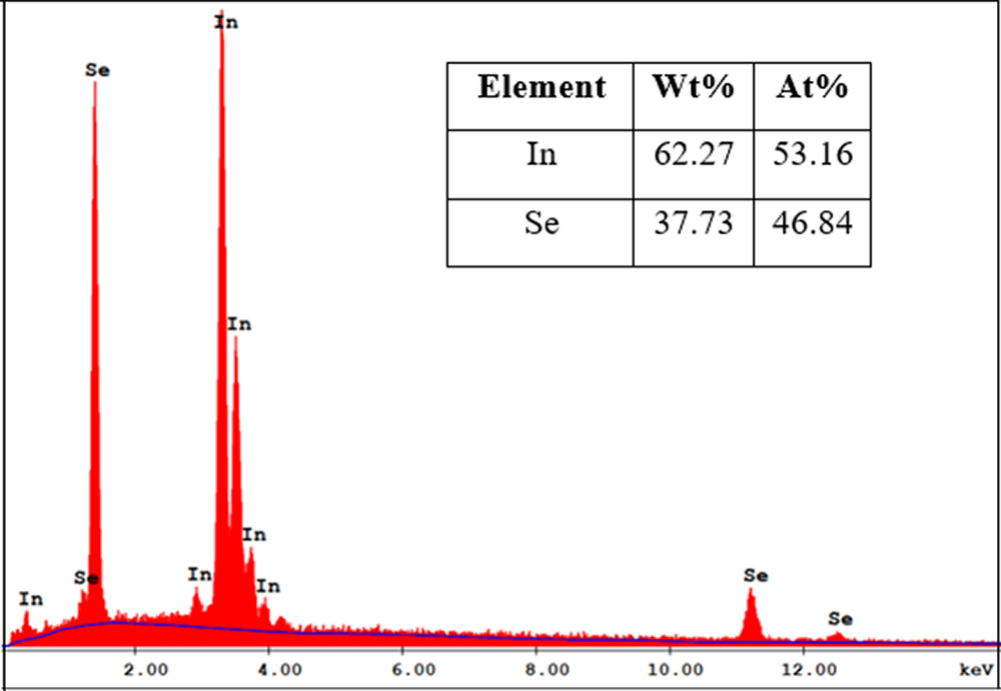
EDX spectrum of the studied InSe thin film.

The studied InSe thin film’s elemental composition was examined by employing EDX. Figure 3 presents the EDX spectrum of the studied InSe thin film. The peaks observed in the spectrum are associated with the indium (In) and selenium (Se) elements’ characteristic emission peaks.^21−23^Figure 3 also shows the atomic composition ratio of the InSe thin film. The analyses of the spectrum revealed that the atomic compositional ratio of In:Se was 53.16:46.84 (53:47). This ratio was found to be acceptable for the chemical formula of the InSe thin film.

**Figure 4:**
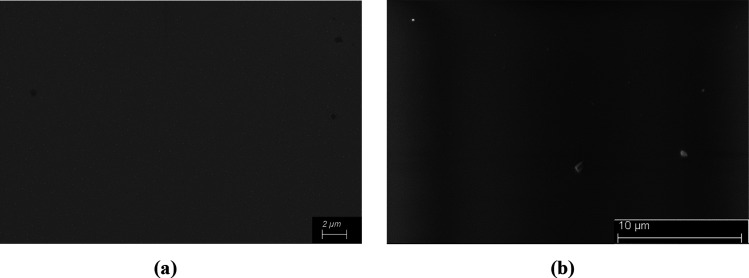
SEM images taken at different magnification values: (a) 10.00 KX and (b) 6.50 KX.

SEM was employed to examine the studied InSe film’s surface morphology. Figure 4 shows SEM images of the InSe film. These SEM images indicate that the InSe film has a uniform and smooth surface.^23,24^

### 3.2 Optical Characterization of the InSe Film

**Figure 5:**
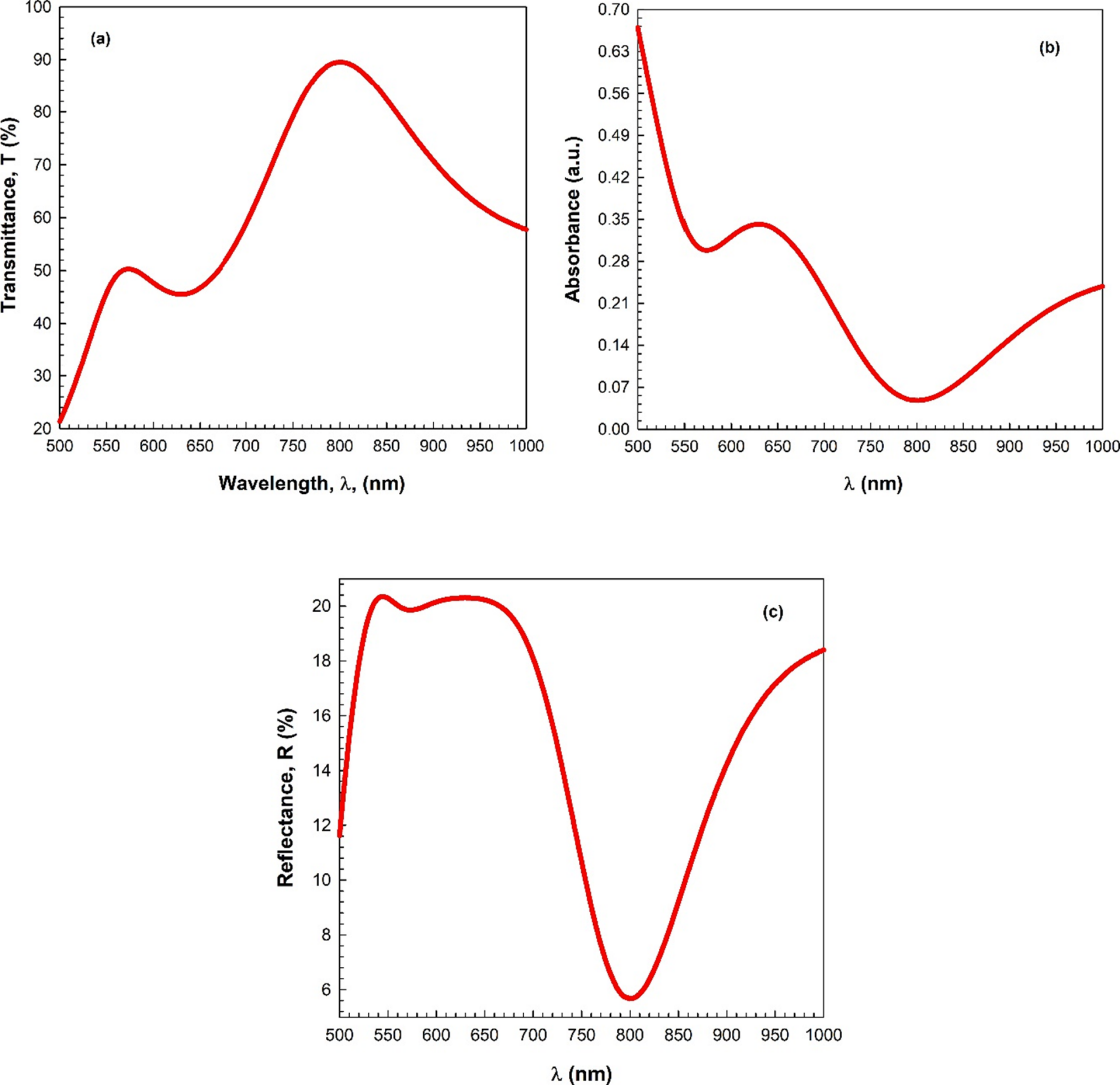
(a) Transmittance, (b) absorbance, and (c) reflectance spectra as a function of the wavelength.

The UV–vis spectroscopy method measures the intensity of light transmitted through or absorbed by a material. In the present study, UV–vis spectroscopy was employed to determine the studied InSe film’s optical properties as a function of wavelength. The optical transmittance (T), absorbance (A), and reflectance (R) spectra are used to examine the optical characteristics of the InSe film. Figure 5a–c shows the studied film’s reflectance, transmittance, and absorbance values, which were measured in the wavelength range between 500 and 1000 nm. According to Figure 5a, the transmittance spectra indicate two peaks observed at the wavelength values of ∼575 and ∼805 nm. The observed decrease in transmittance after the wavelength value of 575 nm, attributed to free-carrier absorption, and the increase in absorbance after the wavelength of about 800 nm in InSe can be explained by the material’s electronic structure and the interactions of photons with charge carriers.

Decrease in transmittance (575 nm): In semiconductors like InSe, free carriers (electrons and holes) can absorb photons when their energy matches the band gap of the material. Beyond the bandgap, the absorption increases, leading to a reduction in transmittance. This is a common phenomenon known as free-carrier absorption. Increase in Absorbance (800 nm): The increase in absorbance at longer wavelengths, specifically around 800 nm, may be attributed to the formation and absorption of free excitons. Free excitons are bound electron–hole pairs, and their absorption spectra typically extend to longer wavelengths. The presence of free excitons can contribute to increased absorbance in this spectral region.

InSe is a layered semiconductor with a direct band gap, and its electronic properties are influenced by quantum confinement effects in thin films. The observed optical behaviors align with the characteristics of layered semiconductors and are consistent with the electronic transitions associated with free carriers and excitons in InSe.

The decrease in transmittance after the wavelength value of 575 nm might be attributed to the free-carrier absorption.^25^ The decrease in transmittance after a wavelength value of 575 nm might be attributed to the free-carrier absorption. Moreover, the InSe film has high transparency in the visible light region and exhibits a high transmittance of about 90%. Figure 5b indicates the variation of the optical absorption with the wavelength. It is clear that the optical absorbance spectrum exhibits low absorbance in the visible region. This low absorption can be related to the defects of the InSe film. The absorption edge of the film is found approximately at a wavelength of 630 nm. Also, the peaks observed in the absorbance spectrum are caused by electronic transitions. Figure 5c demonstrates the variation in the optical reflectance of the studied material with the wavelength. According to this figure, the reflectance shows a broad peak in the visible region. After the visible region, the reflectance rises due to the decline in the energy of the incident photon or radiation. Also, the film shows reflection within the visible range. The T, A, and R values obtained for the studied InSe film are in concurrence with the values in the literature.^14,16,20,26^

**Figure 6:**
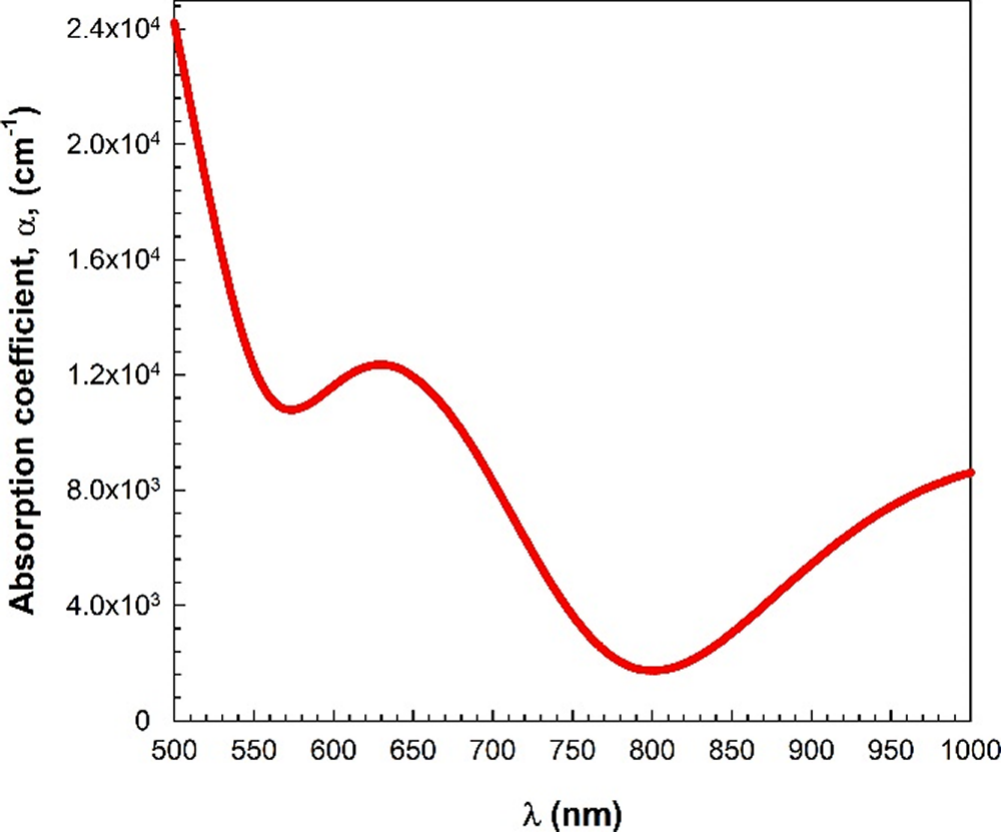
Variation in the absorption coefficient (α) with the wavelength.

The optical constants, including absorption coefficient (α), refractive index (*n*), and extinction coefficient (*k*), were determined using the measured transmittance and reflectance data. As a significant parameter, the absorption coefficient (α) is used to estimate the optical band gap (*E*_g_) of the material. Moreover, the absorption coefficient helps to provide information about the direct and indirect electronic transitions in the material. The transition corresponds to the direct transition for α > 10^4^ cm^–1^ while it corresponds to the indirect transition for α < 10^4^ cm^–1^.^27−31^ The value of the absorption coefficient can be calculated by utilizing the following equation:
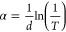
1where d stands for the thickness of the material (about 637 nm for the studied InSe film). Figure 6 demonstrates the variation of α with wavelength (λ). This figure also reveals that the absorption coefficient value is high at low wavelengths (λ < 800 nm or at high photon energies) while it is low at high wavelengths (λ > 800 nm or at low photon energies). This result indicates that the probability of electronic transition at low wavelengths is high. Moreover, the α value is >10^4^ cm^–1^ at low wavelengths. This indicates the possibility of direct transition. The presented InSe film has a direct band gap. This finding is consistent with the literature.^23,34,35^

**Figure 7:**
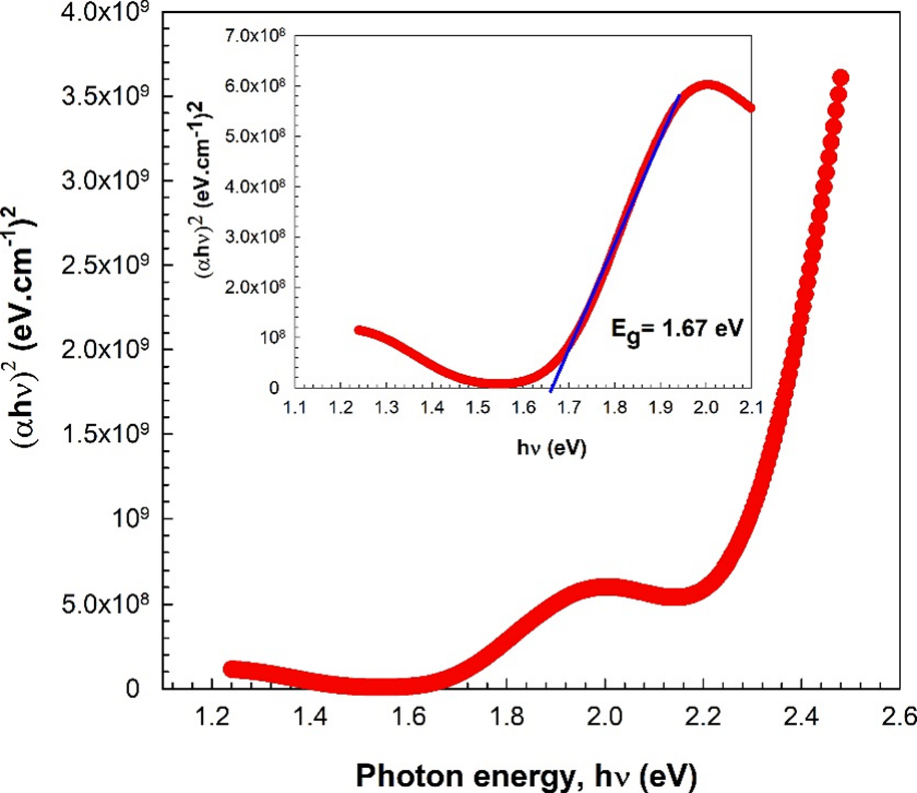
(α*h*ν)^2^ vs photon energy plot.

Tauc’s plot was utilized to obtain the optical band gap (*E*_g_) of the InSe film.^27,36^ The relationship between α and *E*_g_ can be expressed as follows:
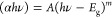
2where *A* stands for the proportionality constant and *h*ν stands for the photon energy. The value of *m* changes, depending on the electronic transition. The value of m is equal to 1/2 or 3/2 for the direct transition, while it is equal to 2 or 3 for the indirect transition. The absorption edge of InSe is described by the direct allowed transition. Figure 7 shows the (α*h*ν)^2^ vs photon energy plot for direct transitions (*m* = 1/2). This plot shows a straight line intercepting the *h*ν axis. The extrapolation of the linear section to (α*h*ν)^2^ = 0 corresponds to the *E*_g_. The studied InSe film was found to have an optical band gap of approximately 1.67 eV. The obtained value was found to be consistent with those reported by the research examining the optical characteristics of InSe.^13,16,20,23,25−28,32−35^

The quantum confinement effect in 2D materials is more appropriately characterized by the reduction in the electronic bandgap as the material’s thickness is reduced. In the context of 2D materials such as InSe, the quantum confinement effect occurs because of the reduced dimensionality. As the material thickness is decreased to a few atomic layers, the electronic states become quantized, and the bandgap increases compared to the bulk material. This effect is a fundamental property of 2D materials.

The specific value of the quantum confinement-induced band gap in InSe would depend on the layer thickness and the material’s properties. It is not determined by the Bohr radius but rather through theoretical and experimental studies of the material’s electronic structure.

Band gaps larger than the bulk value for a thick InSe film suggest that the quantum confinement effect is present even in relatively thick films. This phenomenon can be due to the unique properties of 2D materials, such as InSe^35^

Other optical constants such as the refractive index (*n*) and extinction coefficient (*k*) can be determined by utilizing transmittance and reflectance measurements. The magnitude of *k*, which shows the amount of light lost resulting from absorption and scattering, is calculated as follows^36−39^:
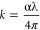
3

On the other hand, the value of the refractive index (*n*) can be computed as follows^20,40−43^:
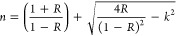
4

**Figure 8:**
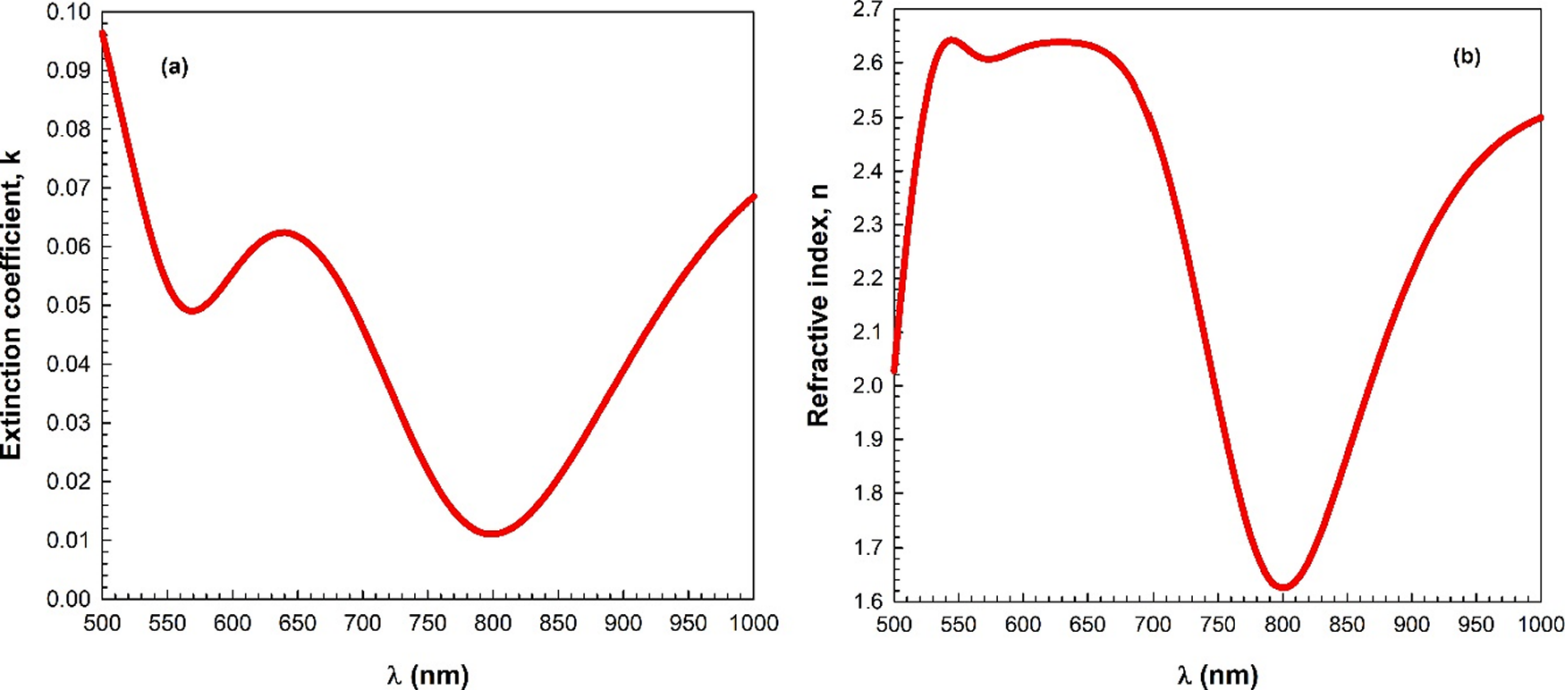
Variation of the (a) extinction coefficient and (b) refractive index with the wavelength.

Figure 8a,b shows the plots of *k* versus wavelength and *n* versus wavelength, respectively. As clearly seen in Figure 8a, the *k*–λ plot has a peak at about 640 nm in the visible region. According to Figure 8b, on the other hand, the *n*–λ plot shows one broad peak in the visible region. At higher wavelength regions (after 800 nm), the values of both *k* and *n* rise with the increase in the wavelength. As a result, *n* gets a greater value than *k* for all wavelengths.

The complex refractive index (n* = *n* + *ik*) can be expressed by using the complex optical dielectric constant (ε*) as follows^43−45^:
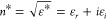
5

where ε_*r*_ and ε_*i*_ represent the real and imaginary components of ε*, respectively. The incident light radiation causes induced polarization in the material. The ε_*r*_ relates to the storage of energy within the material polarized by an electric field while the ε_*i*_ relates to the loss of energy or absorption of energy in the material. The complex dielectric constant, on the other hand, relates to *k* and *n*. The values of ε_*r*_ and ε_*i*_ can be calculated using the following equations, respectively^20,43−47^:
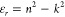
6
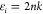
7

**Figure 9:**
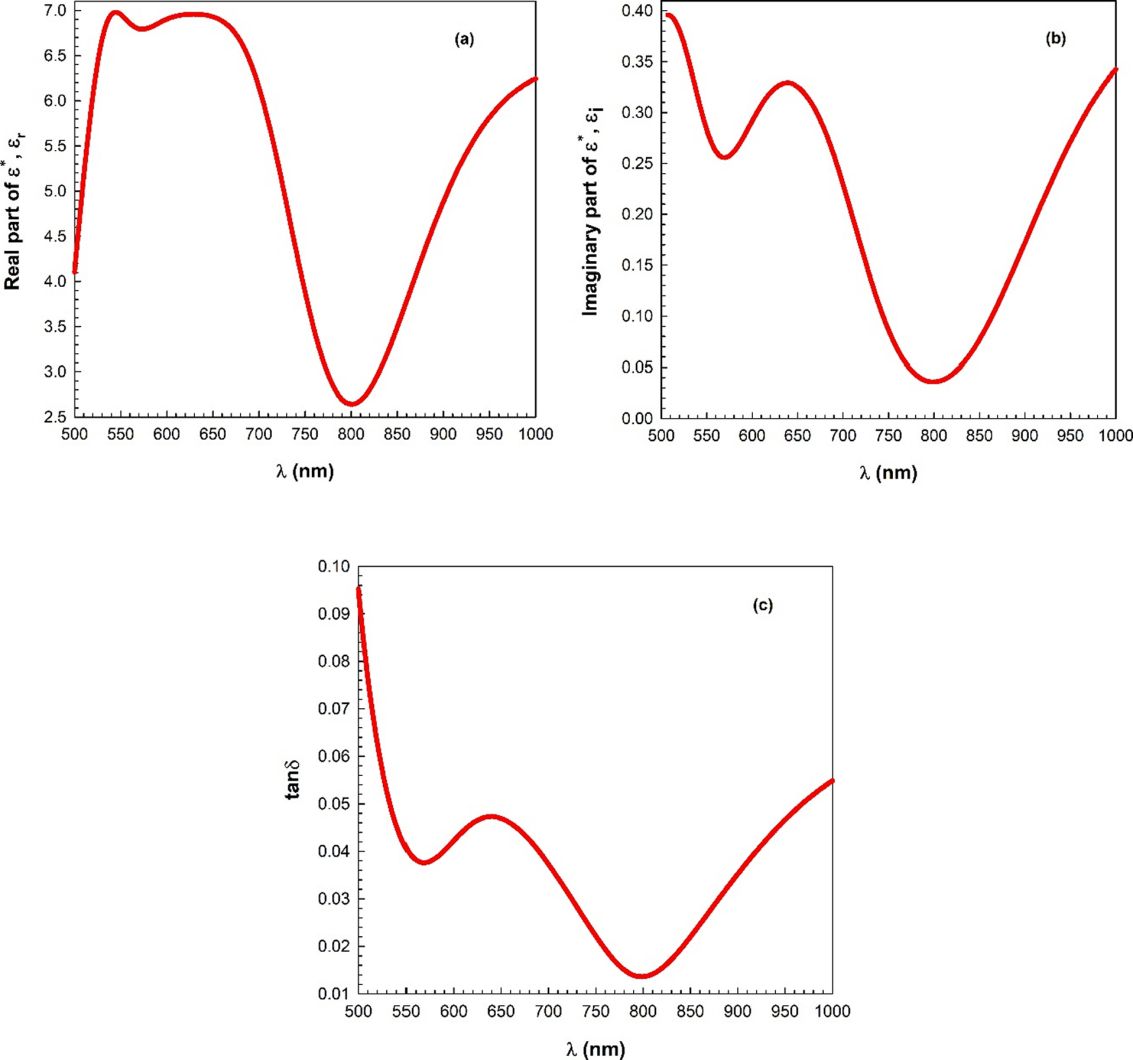
Variation in the (a) real component (ε_*r*_) and (b) imaginary component (ε_*i*_) of the complex dielectric constant (ε*), and (c) dielectric loss tangent (tan δ) with the wavelength.

The real and imaginary components of ε*, namely, ε_*r*_ and ε_*i*_, which are plotted as a function of the wavelength, are shown in Figure 9a,b, respectively. As Figure 9a indicates, the ε_*r*_–λ plot displays a broad peak, similar to the refractive index in the visible region. Figure 9b shows the ε_*i*_–λ plot, which has one peak similar to the extinction coefficient. In addition, both ε_r_ and ε_i_ values increase with an increase in the wavelength after 800 nm. These figures reveal that the ε_*r*_ and ε_*i*_ values are considerably different. So the real part is quite high compared to the imaginary part. Additionally, the ratio of the ε_*i*_ to ε_*r*_ of the complex dielectric constant is defined as the dielectric loss tangent (tan δ). Figure 9c demonstrates the variation in the loss tangent of the InSe film with the wavelength. This figure reveals that the loss tangent declines with the rise in wavelength and it has a peak in the visible region.

**Figure 10:**
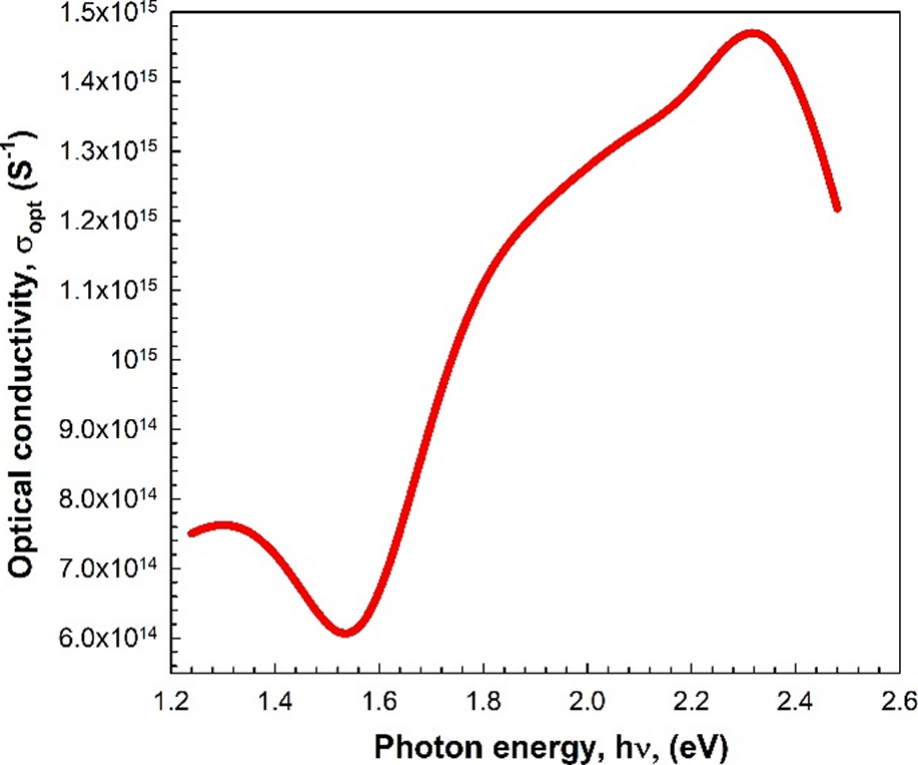
Variation in the optical conductivity (σ_opt_) with the photon energy.

Optical conductivity (σ_opt_) indicates the optical absorption and has a relationship with α and *n*. The σ_opt_ can be expressed as follows^48−52^:
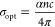
8where *c* stands for the speed of light. Figure 10 presents the variation in the optical conductivity (σ_opt_) with the photon energy. The σ_opt_ value is observed to increase with an increase in the photon energy. High absorption value causes this increase. Also, this indicates that the mobility of electrons increases with the increasing photon energy.

## 4 Conclusions

The optical features and structural characteristics of the InSe film, which was prepared on a glass substrate by using a thermal evaporation method, were extensively studied. The XRD, EDX, and SEM methods were employed to analyze the crystal structure, elemental compositions, and surface morphology of the studied InSe film. Also, the XRD method confirmed the InSe film’s formation. The InSe film has a smooth and uniform surface morphology. The optical constants such as refractive index, absorption coefficient, extinction coefficient, optical band gap, the real and imaginary components of the complex optical dielectric constant, and the optical conductivity were determined in the spectral range between 500 and 1000 nm. The InSe film was observed to have high transparency in the visible light region. In addition, optical analysis indicates that the InSe film has a high absorption coefficient and a direct optical transition. These results reveal that the prepared InSe film has promising potential in 2D optoelectronic device applications.

## Data Availability

The data that support the findings of this study are available from the corresponding author upon reasonable request.
